# Economic evaluation of pharmacogenomic-guided antiplatelet treatment in Spanish patients suffering from acute coronary syndrome participating in the U-PGx PREPARE study

**DOI:** 10.1186/s40246-023-00495-3

**Published:** 2023-06-07

**Authors:** Margarita-Ioanna Koufaki, Vasileios Fragoulakis, Xando Díaz-Villamarín, Kariofyllis Karamperis, Athanassios Vozikis, Jesse J. Swen, Cristina L. Dávila-Fajardo, Konstantinos Z. Vasileiou, George P. Patrinos, Christina Mitropoulou

**Affiliations:** 1 Laboratory of Pharmacogenomics and Individualized Therapy, Department of Pharmacy, University of Patras School of Health Sciences, Patras, Greece; 2 The Golden Helix Foundation, 91 Waterloo Road, Capital Tower 6th Floor, London, SE1 9RT UK; 3 Instituto de Investigación Biosanitaria de Granada (ibs.Granada), Granada, Spain; 4 Laboratory of Health Economics and Management (LabHEM), Economics Department, University of Piraeus, Piraeus, Greece; 5 Leiden University Medical Center, Leiden, The Netherlands; 6 Clinical Pharmacy Department, Hospital Universitario Virgen de las Nieves, Instituto de Investigación Biosanitaria (ibs.Granada), Granada, Spain; 7 Department of Genetics and Genomics, College of Medicine and Health Sciences, United Arab Emirates University, Al-Ain, Abu Dhabi, United Arab Emirates; 8 Zayed Center for Health Sciences, United Arab Emirates University, Al-Ain, Abu Dhabi, United Arab Emirates

**Keywords:** Clopidogrel, Economic evaluation, Cost-effectiveness, Pharmacogenomics-guided treatment, Spain, Acute coronary syndrome, PREPARE

## Abstract

**Background:**

Cardiovascular diseases and especially Acute Coronary Syndrome (ACS) constitute a major health issue impacting millions of patients worldwide. Being a leading cause of death and hospital admissions in many European countries including Spain, it accounts for enormous amounts of healthcare expenditures for its management. Clopidogrel is one of the oldest antiplatelet medications used as standard of care in ACS.

**Methods:**

In this study, we performed an economic evaluation study to estimate whether a genome-guided clopidogrel treatment is cost-effective compared to conventional one in a large cohort of 243 individuals of Spanish origin suffering from ACS and treated with clopidogrel. Data were derived from the U-PGx PREPARE clinical trial. Effectiveness was measured as survival of individuals while study data on safety and efficacy, as well as on resource utilization associated with each adverse drug reaction were used to measure costs to treat these adverse drug reactions. A generalized linear regression model was used to estimate cost differences for both study groups.

**Results:**

Based on our findings, PGx-guided treatment group is cost-effective. PGx-guided treatment demonstrated to have 50% less hospital admissions, reduced emergency visits and almost 13% less ADRs compared to the non-PGx approach with mean QALY 1.07 (95% CI, 1.04–1.10) versus 1.06 (95% CI, 1.03–1.09) for the control group, while life years for both groups were 1.24 (95% CI, 1.20–1.26) and 1.23 (95% CI, 1.19–1.26), respectively. The mean total cost of PGx-guided treatment was 50% less expensive than conventional therapy with clopidogrel [€883 (95% UI, €316–€1582), compared to €1,755 (95% UI, €765–€2949)].

**Conclusion:**

These findings suggest that PGx-guided clopidogrel treatment represents a cost-effective option for patients suffering from ACS in the Spanish healthcare setting.

## Introduction

Cardiovascular diseases (CVDs) are one of the leading causes of mortality all over the world. Being classified as a non-communicable disease, CVDs are a dominant health issue with both social and economic burdens. In numbers, CVDs are the leading cause of death in Spain accounting for almost 120.000 deaths per year while it is the second reason for hospital admissions estimating to reach almost 592.000 hospitalizations per year [1–3]^.^

Acute coronary syndrome (ACS) constitutes a life-threatening CVD type, associated with high risk of morbidity and mortality. It includes a range of heart conditions related to sudden, reduced blood flow to the heart. Myocardial infarction (MI) (both ST elevation myocardial infarction (STEMI) and non-ST (NSTEMI)) and unstable angina are a few examples of ACS [4]. Unfortunately, ACS incidence rate is rapidly increasing all over the world due to modifiable factors such as smoking, obesity, extensive alcohol consumption, diabetes mellitus, hypertension, etc. [5, 6]. Therapy with antiplatelets is the first-line treatment strategy for ACS since dual antiplatelet therapy with aspirin and a P2Y12 receptor antagonist is usually recommended [7].

Clopidogrel is a well-known P2Y(12) receptor antagonist commonly prescribed to ACS patients with high bleeding risk [7, 8]. This antiplatelet prodrug is metabolized by CYP450 in liver to active metabolite (clop-H4) that inhibits platelet aggregation and subsequent thrombogenesis by binding to ADP platelet receptor P2Y12 [9, 10]. Less than 15% of the prodrug is transformed into an active form, while the remaining 85% is hydrolyzed by esterases to inactive forms, subsequently excreted from the human body [11, 12]. CYP2C19 enzyme is encoded from the *CYP2C19* gene, which is highly polymorphic, and it shows great variability (approximately 12%) among populations due to inter-individual and inter-ethnic differences in the genetic background, resulting in significant variation in the drug metabolizing status of the CYP2C19 enzyme, both in terms of drug efficacy and toxicity [13, 14]. Being involved in the whole bioactivation process of clopidogrel, *CYP2C19* genetic variation exerts a significant impact on the formation of active metabolite.

Indeed, approximately 5–40% of patients treated with conventional doses of clopidogrel display inadequate antiplatelet responses owing to low inhibition of ADP-induced platelet activation, which could lead to severe cardiovascular and cerebrovascular complications [7, 13]. Evidently, such phenomena are mainly attributed to genetic variants in *CYP2C19*, resulting in poor or intermediate metabolizer phenotypes (PM and IM) that are receiving a sub-optimal therapy and have a high on treatment platelet reactivity [14, 15]. Many randomized controlled clinical trials like POPular Genetics and TAILOR-PCI have established the correlation between *CYP2C19* genotype and clopidogrel response primarily in the cohort of ACS patients undergoing PCI showing the importance of de-escalation of dose based on genetic results and switching to different P2Y12 receptor antagonist, a fact that highlights the potential impact of PGx testing in antiplatelet treatment [8, 16].

Clopidogrel is one of the first medications to be associated with pharmacogenomic (PGx) biomarkers and clinical guidelines [17]. Based on the Dutch Pharmacogenomics Working Group (DPWG), patients with an actionable phenotype due to a genetic variation in the *CYP2C19* gene are recommended to either undertake higher drug dosage (IM) or switch to an alternative antiplatelet therapy (PM) (e.g., prasugrel or ticagrelor) in case of no other contraindication to avoid adverse drug reactions [18].

Antiplatelet treatment and P2Y12 receptor antagonists have high risk of severe ADRs that can lead to a person’s hospitalization and are commonly related with severe bleeding events [7, 13]. In accordance with the latest European estimations, the annual CVDs cost to European Union economy can reach up to 210 billion euros per year and 53% of those are related to healthcare costs due to hospitalization [19]. Hospitalization costs along with high incidence of ADRs, population aging, and scarcity of available resources put viability of European healthcare systems at risk. PGx is a promising technology that can improve the overall flow of drug and disease management by tailoring one’s medication according to individual’s genomic profile, and consequently to reduce the risk of ADRs and at the same time maximize treatment’s efficacy [20]. Evidently, PGx-guided strategy in antiplatelets is shown to bring fruitful results in ACS disease management (reduced MACE, bleeding events) in accordance with several randomized clinical trials and it has been characterized as a “reasonable alternative for standard P2Y12 inhibitor therapy based on European Society of Cardiology guidelines published in 2021 [7, 8, 16]. By taking into consideration the disease prevalence and incidence, even a small improvement thanks to the adoption of PGx-guided treatment is likely to be translated into meaningful population-level health gains [21]. Nonetheless, physicians treating CVD patients haven’t widely adopted this initiative and PGx strategy hasn’t been implemented in the clinical setting [20].

Given that the available resources are rather scarce, the aim of the present study is to estimate whether a PGx-guided clopidogrel treatment is cost-effective compared to conventional clopidogrel treatment in patients diagnosed with ACS in the Spanish healthcare setting.

## Methods and materials

### Data collection

Both clinical and economic data derived from the PREPARE (PREemptive Pharmacogenomic testing for preventing Adverse drug REactions study), a prospective, open-label, randomized controlled clinical trial having taken place at the University Hospital of San Cecilio, the University hospital Virgen de las Nieves, the Zaidin South Primary Care Centre, and the Zaidin Specialty Centre, Granada Spain, from May 2017 until June 2020 [22]. PREPARE is the first and largest multinational, open-label, controlled, cluster-randomized, crossover implementation study that investigates the clinical and cost-effectiveness of implementing preemptive genotyping testing in the population using a PGx panel [23, 24]. PREPARE protocol was previously reported elsewhere [23, 24]. The present analysis refers to data collected from Spanish sites participating in the study. The study analysis was undertaken based on 243 participants for both arms, 113 subjects in the PGx group and 130 in the control group, for whom detailed medical records were documented in source documents and in study’s electronic case report system (eCRF).

### Study design

All inclusion and exclusion criteria of the study are briefly described below. Subjects of any ethnicity, ≥ 18 years of age with a clinical diagnosis of a type of ACS (i.e., MI, unstable angina, ST and non-ST elevation, STEMI and NSTEMI) that were primer naïve to clopidogrel, hadn’t undertaken any genetic testing in the past for *CYP2C19*, consented to be followed up for at least 12 weeks and could give blood or saliva sample were eligible to participate in the study. Patients were excluded in case that (a) they were reluctant to give signed informed consent, (b) were pregnant or breastfeeding, (c) were suffering from advanced liver failure (stage Child–Pugh C) or had an existing impaired hepatic or renal function, (d) their estimated life expectancy was less than 3 months and (e) had no fixed address or an assigned general practitioner. Physicians participating in the study established the diagnosis of ACS, the life expectancy of patient and medical history of each patient relying on all available clinical data [24].

In Spain, PGx-guided treatment group run from April 2017 until September 2018 and the other group from November 2018 until June 2020. All study participants were followed up for a minimum of 12 weeks and no more than 18 months. Control group followed a non-tailored treatment strategy based on the common clinical routine related to clopidogrel whereas PGx-guided group received a PGx-guided treatment strategy based on each patient’s *CYP2C19* genotyping results. During the study, subjects were asked to complete two online questionnaires at week 2 and at week 8 and to perform four interviews called nurse assessments on baseline, week 4, week 12 and upon 18 months. Those nurse assessments were conducted either via phone calls remotely or on-site interviews by trained research personnel and included questions about disease progression, subject’s quality of life, the occurrence of any adverse event, use of any concomitant medication or procedure and any hospitalization event.

On baseline visit, well-trained physicians discussed with participants all study’s requirements including saliva sample, follow-up visits, and interviews and provided them with the informed consent form. Upon giving informed consent, patients donated saliva sample were randomized and prescribed clopidogrel in 75 mg/per day as loading dose. Genetic results of PGx group were available within 7 days upon sample collection day. Then, physicians reviewed each patient’s results to tailor individual’s clopidogrel treatment either by adjusting the dosage or by changing medication in accordance with DPWG relevant guidelines [18]. Therefore, PGx treatment strategy and maintenance dose were finalized a week upon patient’s enrolment.

Basic participants’ demographics information including gender, age, body-mass index (BMI), smoking and alcohol consumption status along with clinical data such as comorbidities and co-medication use was recorded at the baseline visit (see Table 1). Data related to adverse events, utilities, visits to emergency units, and hospital admissions were collected via the nurse assessments as mentioned above.

**Table 1:** Patient’s characteristics

	PGx-guided group	Control group
*Gender (%)*		
All	113	130
Male	62 (54.8%)	75 (57.6%)
Female	51 (45.1%)	55 (42.3%)
*Indication*		
STEMI	13	13
NSTEMI	52	0
ACS	3	33
Heart failure	3	10
Unstable angina	13	11
PCI	5	0
MI	4	60
Catheterization	7	0
Chest pain	3	0
Coronariography	5	0
Coronary Artery Disease	5	0
Aortic Stenosis	0	3
*Genotype*		
All	113	130
Wild-type	47	60
Poor Metabolizer	5	2
Intermediate Metabolizer	20	30
Extensive metabolizer	34	38
Ultra-metabolizer	7	0
*Age (SD)*		
All	74 (11.8)	78 (10.2)
Male	71 (13.5)	76 (10.1)
Female	76 (8.6)	81 (9.9)
*BMI (SD)*		
All	27,64 (4.4)	27,98 (4.3)
Male	27,19 (4.6)	27,77 (3.8)
Female	28,19 (4.2)	28,27 (4.8)
Smoking (%)		
Non-Smoker	60 (53%)	71 (54.6%)
Previous smoker	45 (39.8%)	43 (33%)
Current smoker	8 (7%)	16 (12.3%)
Alcohol consumption %*		
All	21	30
Male	16 (76.1%)	30 (100%)
Female	5 (23.8%)	0
Diabetes (%)**		
All	45	55
Male	24 (53.3%)	38 (69%)
Female	21 (46.6%)	17 (30.9%)
*Hypertension (%)*		
All	75	20
Male	39 (52%)	10 (50%)
Female	36 (48%)	10 (50%)

*Alcohol consumption refers to > 3 drinks per day

**Cases of type 2 diabetes mellitus were taken into consideration

*SD* Standard Deviation

All available data of the present analysis were collected by clinical staff trained in study’s protocol and systems. Data were reviewed and reconciliated by two of the main authors of the paper for any typos or discrepancies between source documents and eCRF. Upon reviewing database, 243 patients were included in the analysis. PREPARE trial was performed in compliance with the 1964 Helsinki declaration. It was approved by Comité Coordinador de Ética de la Investigación Biomédica de Andalucía (CCEIBA)—ethics committee in Spain [25], and it is registered on clinicaltrials.gov (NCT03093818).

### Perspective of analysis

The analysis perspective of this study was that of sickness fund [26]. All type of direct medical costs (hospitalization costs, emergency costs, follow-up costs, genetic testing cost) along with the relevant induced costs were included. Those costs were reimbursed by the payers in Spanish Prefecture of Andalusia. Other direct costs borne and paid by the patients (diet costs, travel expenses, home nurse aide, etc.) or indirect costs such as loss of productivity due to absenteeism [27], albeit important, were not taken into consideration for this analysis.

### Missing data analysis

Dealing with missing data is a common issue in economic analysis, and their proper handling might improve the cost-effectiveness conclusions [28]. Following Faria and coworkers, a descriptive analysis was undertaken to provide details regarding the percentage of missing values in individuals’ answers in nurse assessments including both details about utilities and assessment dates [29]. Then, a logistic regression was run to gain insight into the association among missingness—which represented as a binary variable—and (a) baseline characteristics (such as age, gender, BMI, etc.) and (b) final outcomes (total cost and quality-adjusted life years (QALYs)) [29–31].

Missing baseline values can have a great impact on the analysis, on the ground that it might be necessary to use those missing values to predict subsequent outcomes [29]. Indeed, single imputation method was applied for baseline utility in each treatment arm, by filling the missing values with the average of the observed cases [24]. For intermittent missing data in quality-of-life answers, when possible, linear interpolation method was used between measurement points [32, 33], while multiple imputation method with five imputed datasets was done for the rest of them [34–36].

### Right censored cost data

Right censoring for cost data is a specific case of missingness in which some individuals are lost to follow-up within the study period or still alive at the time of study completion and, thus, their complete/total costs are not available for statistical analysis [35]. To deal with this issue, the nonparametric, unbiased and consistent Bang–Tsiatis estimator was employed [36, 37], adding a correction term to improve efficiency (Zhao-Tian estimator). In short, this estimator calculates the weighted cost for each uncensored individual per group, based on the inverse probability of being censored at the time of failure. For computational purposes, a more intuitive replace-from-the-right algorithm was used as an equivalent alternative to the Zhao-Tian estimator [38]. Briefly, the cost of each censored individual was replaced by the average of costs of those individuals who survived longer than him/her, taking also into account the mean cost of each censored patient into account at the time of censoring and projecting this cost to the estimated unobserved survival [39–45].

### Utility values

Utility values describe the health-related quality of life (QoL) associated with different health states. In the original analysis plan, time-trade-off method was applied, but this plan was abandoned due to the low response rate of participants [46]. Hence, utility weights were also extracted from the literature [6, 47–52]. In particular, the ‘‘well’’ state was set at 0.87, while for those experiencing any major event, utility decrements and the correspondence duration were used (see Table 2). In sensitivity analysis, the QoL was estimated by means of participants’ VAS score given at baseline visit, week 4, week 12 and 18 months from baseline. Quality-adjusted life years (QALYs) were measured by calculating the integral of the product of individual’s life expectancy multiplied by weighted VAS score and adjusting the baseline measures of utility within a covariate regression framework [53].

**Table 2:** Calculation of utility decrements

	Duration*	Utility	Calculation	Description
Acute Coronary Syndrome G5	30	0.68	0.87 − 0.19	[47, 48, 52]
Acute Coronary Syndrome G4	30	0.68	0.87 − 0.19 + 0.02	Assumption based on similar studies
Heart Failure G5	90	0.56	0.87 − 0.31	[49]
Heart Failure G4	90	0.56	0.87 − 0.31 + 0.02	Assumption based on similar studies
Cardiac Arrest G5	90	0.609	0.87 − 0.261	[49, 50]
Stroke G4	180	0.64	–	[49]
Gastric Hemorrhage G4	30	0.696	0.87 − 0.174	[50]
Chest Pain G3	30	0.69	–	[49]
Myocardial Infarction G3	180	0.75	–	[49]
Atrial Fibrillation G3	90	0.65	–	[49]
Rectal Hemorrhage G3	30	0.696	0.87 − 0.174	[50]
Gastrointestinal Pain	30	0.68	–	Derived from the model
Colonic Hemorrhage	30	0.696	0.87 − 0.174	[50]
Dizziness	30	0.68	–	[51]
Diarrhea	30	0.72	–	[52]
Oral Hemorrhage	30	0.696	0.87 − 0.174	[50]
Baseline	–	0.87	–	[6, 47]

*Duration is estimated in days based on the literature and on the experts' opinion; G indicates grade

### Costing methodology and economic analysis

Treatment’ effectiveness was determined by mean survival and it was estimated based on the official start date of clopidogrel to (a) death related to the CVDs (complete cases), (b) death from any cause (complete cases), (c) loss to follow-up (censored patients) or (d) to the end of study period (censored patients). Total cost included (a) the cost of ADRs, (b) hospitalization’s costs, (c) follow-up costs and (d) the cost of genetic testing applicable only for PGx-guided group. Cost of index drug (clopidogrel) itself was not taken into account in the analysis owing to the fact that both groups represent a pool of individuals with different health status and comorbidities and only ADRs’ cost can make a difference. Similarly with a previous pharmacoeconomic analysis [54], patient-level resource utilization data were combined with unit cost data and then aggregated to compute total treatment cost per patient. The following ADRs were considered for cost evaluation: ACS, gastrointestinal pain, heart failure, dizziness, chest pain, cardiac arrest, stroke, MI, atrial fibrillation, diarrhea, cardiac arrest, oral hemorrhage, rectal hemorrhage and colonic hemorrhage (see Table 2). Variation in resource utilization among individuals reflects differences related to hospitalization, health complications, unplanned operations, laboratory tests, etc. Reimbursement tariffs used were obtained from the official sources [55] and were applicable to all public hospitals and public payers of Andalucía community region in Spain. All components’ costs are presented in Table 3. Due to limited time horizon of this observational study, discount rate was not applied. In addition, due to lack of official price, genetic test’s cost was extracted from the literature [61] and is consistent to those reported in another Mediterranean country (Italy) [51].

**Table 3:** Cost per item used in the economic analysis

Medical Interventions	Cost €
Hospitalization events**	
Amputation	9566.45
Gastrointestinal bleeding	3065.25
Urethra procedures	2767.29
Coronary angioplasty	7476.26
Surgery	3431.18
Urinary tract infection	3977.88
Pacemaker	13.955
Chest Pain	2002.72
Thrombectomy	2780.82
Transcatheter Aortic Valve Implantation	2578.81
Cerebrovascular Problems	7506.23
Anemia	3048
Fever	2810.64
Respiratory disease	4612.76
Catheterization	2404.29
Angina pectoris	3306.27
Colon adenocarcinoma	8111.91
Respiratory Infection Without Surgery	2384.45
Syncope	2574.40
Circulatory Disorder with catheterization without Acute Myocardial Syndrome	2404.29
Atrial Fibrillation	2929.31
Total colon examination	1734.25
Fibrinolysis	6803.55
Infection of urine tract	3977.88
Heart Failure	5599.23
Acute renal failure	5432.46
Intensive care	5599.23
Non-ST elevation myocardial infarction	6891.76
Cardioversion	24,830.64
Atrial Flutter	2929.31
Colitis Ulcerosa	3306.27
Νon-ST-Segment elevation acute Coronary Syndrome	20,675.28
Sepsis	6669.48
*Instrumental examination costs*	
Radiography	9.23
Electrocardiogram	16.30
X-ray Abdomen	9.23
Colonoscopy	81.15
Computerized Tomography Angiography	361.96
Rhinoscopy	161.55
Eco Doppler	92.30
Crossmatch	17.21
Blood transfusion	3891.17
PT/INR	9.54
Abdominal Ultrasound Exam	36.92
Gammagraphy	193.24
Endoscopy	631.47
Echocardiography	36.92
Thoracentesis	81.15
*Laboratory exams*	
Blood test	43.72
Troponin	23.30
Urine test	17.47
Flu PCR	7.94
Gas blood test	3.29
Blood culture	19.99
*Genetic testing*	
PGx testing	120

^*^All costs data derived from Andalucian Regional Health System  [55]

^**^It refers to all procedures done due to adverse event and adverse drug reactions.,

*PCR* Polymerase Chain Reaction, *PT/INR* Prothrombin Time/International Normalized Ratio

Finally, a generalized linear model (GLM) was employed to estimate the effect of covariates (patients characteristics) in total cost to achieve greater flexibility in the presence of heteroskedasticity and right skewness in cost data [56]. In particular, a tweedie distribution was assumed for cost and a logarithmic as a link function. Moreover, a multivariate seemingly unrelated regression equation was employed to provide the necessary information for statistical inference in cost-effectiveness analyses, namely differences in costs and QALYs along with the correlation between the estimations [51]. Incremental Cost-Effectiveness Ratio (ICER) was determined as the ratio of the difference in costs between PGx-guided group vs control group divided by the difference in QALYs.

### Uncertainty

A probabilistic sensitivity analysis was undertaken to test data robustness and to identify how the deterministic results vary under uncertainty [54]. In particular, a new dataset with 5000 nonparametric bootstrap replications with replacement was constructed to determine confidence intervals for the main variables. In the present analysis, the straightforward percentile method was applied [57, 58]. Cost-Effectiveness Acceptability Curve was used to illustrate probabilistic results, which shows the probability (on the y-axis) that PGx-guided group may be cost-effective compared to control group for a range (on the *x*-axis) of maximum monetary values that a decision-maker might be willing to pay per QALY. Based on the assumption of bivariate normality, an ellipse and its contour were constructed to represent the 95% confidence intervals [59]. As a last step, a Value of Information Analysis was performed to investigate the monetary value that can be adjusted to eliminate uncertainty in the decision-making process [60]. The main metrics used were the Expected Value of Perfect Information (EVPI) value, for three different willingness-to-pay thresholds for a QALY, except of partially EVPI.

## Results

The number and proportion of complete data in each nurse assessment are shown in Table 4. In addition, Table 5 summarizes the multiple logistic regression model which explore the relationship between the presence of censoring and baseline characteristics. The log of the odds of a censored case was found to be positively associated with hypertension (OR 0.20, 95% CI: 0.05–0.75, *p* value = 0.017). The Hosmer & Lemeshow (H–L) goodness of fit test was estimated at *x*^2^ (8) = 12.24, *p* = 0.141, while the Nagelkerke (pseudo) *R*^2^ was 17.6%. The overall predictive score of the model was very high, estimated at 94.2%.

**Table 4:** Number and proportion of nurse assessments with complete data

Complete at nurse assessment on	Date of assessment (*n* = 243)	Utility values (*n* = 243)
Baseline Visit	243 (100%)	243 (100%)
Week 4	241 (99.1%)	239 (98.3%)
Week 12	238 (97.9%)	234 (96.2%)
18 months	163 (67%)	160 (65.8%)

**Table 5:** Association of the presence of censoring and baseline characteristics

Parameter	B	S.E	95% LCI	95% UCI	Exp (B)	*p* value
Study arm	− 1.933	0.776	0.032	0.663	0.145	0.13
Gender	− 0.046	0.765	0.213	4.275	0.955	0.955
Age	− 0.050	0.037	0.884	1.023	0.951	0.951
BMI	− 0.088	0.077	0.939	1.271	1.092	1.092
Smoking	0.610	0.535	0.645	5.249	1.841	1.841
Alcohol	0.320	0.412	0.614	3.088	1.377	1.377
Hypertension	− 1.601	0.673	0.054	0.754	0.202	0.202
Diabetes	− 0.554	0.606	0.175	1.883	0.575	0.575

In contrast, analysis indicated that the association of censoring with final outcomes (total cost and QALYs) were not statistically significant when adjusted for baseline characteristics (not shown in tables, available on request). Thus, it was concluded that there was a Missing-at-Random covariance-depended context and the correction of censoring was applied separately for those suffering from hypertension in each group. In a similar manner, missing data were also covariance-dependent, and consequently, a missing-at-random hypothesis was adopted for the multiple imputation analysis (see Tables 6 and 7).

**Table 6:** Main results of the analysis

	Emergency visits (%)	Hospitalization days	Adverse events (%)
*PGx-guided group*			
B-Mean	11.56	0.39	0.15
B-Stdev	2.98	0.20	0.03
B-Max	23.89	1.42	0.29
B-Min	1.77	0.00	0.04
95%UCI	17.70	0.84	0.22
95%LCI	6.19	0.05	0.09
*Control group*			
B-Mean	13.25	0.88	27.94
B-Stdev	2.97	0.31	3.96
B-Max	25.38	2.13	43.08
B-Min	3.08	0.01	14.62
95%UCI	19.23	1.53	35.38
95%LCI	7.69	0.35	20.00
*Control versus PGx-guided*			
B-Mean	1.69	0.49	12.82
B-Stdev	4.26	0.36	5.23
B-Max	18.00	1.85	30.50
B-Min	− 13.55	− 0.81	− 5.55
95% UCI	10.07	1.23	23.11
95% LCI	− 6.55	− 0.21	2.72

Results were based on 5000 bootstrap experiments

*B* Bootstrap, *LCI* Lower Confidence Interval, *PGx* Pharmacogenomic, *SD* Standard deviation, *UCI* Upper confidence interval

**Table 7:** Cost differences (€) between pharmacogenomics and non-pharmacogenomics groups per patient

	Emergency unit cost	Hospitalization cost	Follow-up cost	Total cost*
*PGx-guided group*				
B-Mean	117.8	574.0	70.7	882.5
B-Stdev	39.2	283.1	8.3	324.0
B-Max	286.6	1877.3	103.6	2355.7
B-Min	9.3	0.0	42.9	184.4
95%UCI	200.8	1187.5	88.4	1581.8
95%LCI	46.8	75.7	56.1	315.9
*Control group*				
B-Mean	298.8	1367.6	89.0	1755.4
B-Stdev	115.9	462.4	32.9	556.0
B-Max	865.6	3250.1	234.2	4128.2
B-Min	21.6	105.2	16.8	143.6
95%UCI	564.6	2350.2	164.4	2949.0
95%LCI	115.6	554.3	36.4	765.1
*Control v/s PGx-guided group*				
B-Mean	181.0	793.6	18.3	872.9
B-Stdev	122.3	543.0	34.1	644.4
B-Max	758.3	2887.1	159.8	3518.4
B-Min	− 154.8	− 1277.4	− 60.0	− 1556.0
95% UCI	454.5	1914.9	95.2	2188.7
95% LCI	− 24.7	− 249.0	− 38.1	− 356.8

Results were based on 5000 bootstrap experiments

*B* Bootstrap, *LCI* Lower Confidence Interval, *PGx* Pharmacogenomics, *SD* Standard deviation, *UCI* Upper confidence interval

*PGx-guided group incorporates the cost of genetic testing (€120) in total cost

In general, PGx-guided group shared better results in several parameters. More precisely, it was found that PGx-guided group was associated with fewer visits in emergency units, less ADRs and fewer hospital admissions compared to the control group and subsequently, lower costs. However, no statistically significant difference was found between the groups in terms of QALYs and life-years (LYs) The mean estimate for QALYs (base case scenario) in the PGx-guided group was 1.07 (95% CI, 1.04–1.10) versus 1.06 (95% CI, 1.03–1.09) for the control group, while LYs for both groups were estimated at 1.24 (95% CI, 1.20–1.26) and 1.23 (95% CI, 1.19–1.26), respectively.

PGx-guided group shared better results in terms of VAS score. In particular, PGx-guided group shared 0.84 QALYs (95% CI, 0.80–0.88) in comparison with 0.76 QALYs (95% CI, 0.72–0.79) of the control group. Furthermore, the mean total cost of PGx-guided group was €883 (95% UI, €316–€1582), while control group shared a mean cost of €1755 (95% UI, €765–€2949), a finding suggesting that PGx-guided treatment might be a cost-saving option with a mean difference of €873 (95% CI, €− 389–€2189).

Furthermore, and importantly, health utilization costs were much less (35.05%) in the PGx-guided group (8408.44 EUR) compared to the control group (12,939.29 EUR; see Table 8). Hospital admission costs accounted for most of the expenses in both groups (65% in the PGx-guided group versus 77.9% in the control group) followed by emergency units (13.3% in the PGx-guided group versus 17% in the control group) and follow-up costs (2.8% in the PGx-guided group versus 5.1% in the control group. PGx-guided group had an additional cost dedicated to genotyping testing that represented a 13.6% of the total group’s costs. It is noteworthy that there were a few extreme values because some costs in patients of both groups were as low as €130 or even lower, while others as much as €12,000 or even more due to more expensive resources consumed to deal with their adverse events.

**Table 8:** Data on Health Utilization costs (*n* EUR)

	Visits to emergency unit	Emergency visit costs*	Hospitalization Days**	Hospitalization costs*	Follow-up visits	Follow-up costs*	Genetic testing costs*
PGx-guided arm	13	664.30	9	7522.49	15	101.65	120
Control arm	17	1146.65	11.3	11,593.33	24	199.31	0

^*^Average cost per arm, ** Average number of hospitalization days

**Fig. 1:**
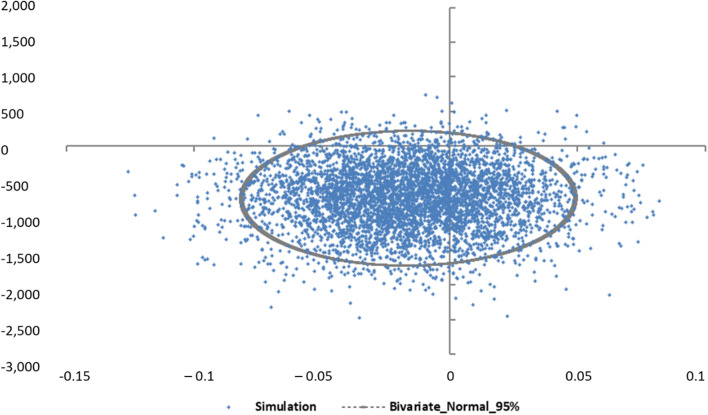
Scatter plot of probabilistic analysis (PGx-guided group vs control). Ellipse represents the 95% uncertainty intervals

The results of the GLM illustrated in Table 9 highlighted that variables such as study group, diabetes and hypertension were statistically significant, while the rest of variables didn’t provide any additional predicted value to the model and thus, were excluded from the analysis. Based on parameters’ estimates for seemingly unrelated regression model, the main cost-effectiveness parameters were: (ΔC = €1229.3 ± 566.7, ΔΕ = − 0.199 (572.4) and *r* = 0.001). Since the standard deviations (SD) were high for both groups, with very low correlation coefficient, a nonparametric bootstrap replication was preferred. In particular, Fig. 1 depicts the joint distribution of 5,000 bootstrap experiments of the difference in the total cost and in the effectiveness (measured in QALYs), between the two study groups.

**Table 9:** Association of the ADRs costs with study groups and study participants’ characteristics

Parameter	B	95% LCI	95%UCI	Exp(B)	95% LCI	95% UCI	*p* value
(Intercept)	3.689	1.324	6.054	39.99	3.757	425.62	0.002
Study arm	0.908	0.348	1.469	2.48	1.416	4.344	0.001
Gender	0353	− 0.193	0.9	1.424	0.824	2.459	0.205
Smoking	0.282	− 0.187	0.751	1.326	0.83	2.119	0.238
Hypertension	0.662	0.12	1.203	1.939	1.128	3.332	0.017
Diabetes	1.593	1.152	2.034	4.917	3.163	7.644	0.000
Age	0.001	− 0.023	0.023	1	0.977	1.023	0.992
BMI	− 0.015	− 0.064	0.033	0.985	0.938	1.034	0.536
(Scale)	87.652^a^	77.35	99.32				

Dependent Variable: Total Cost; Generalized Linear Model; Tweedie distribution with log-link function; Model: (Intercept), Study arm, Gender, Smoking, Hypertension, Diabetes, Age, BMI

^a^Maximum likelihood estimate

*LCI* Lower Confidence Interval, *UCI* Upper Confidence Interval

**Fig. 2:**
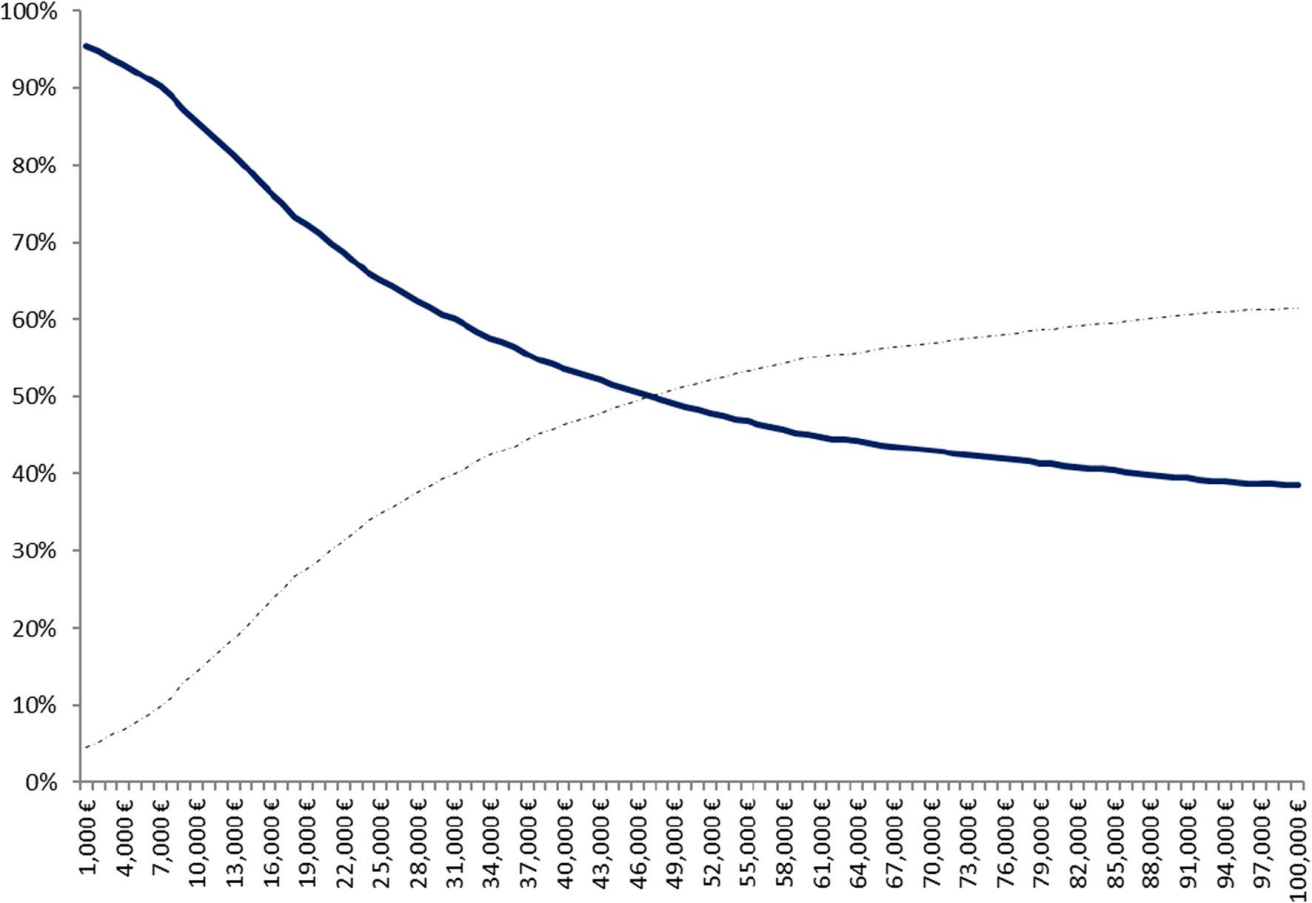
Cost-effectiveness acceptability curve of PGx-guided group vs control. *Y*-axis represents the probability of PGx technology to be cost-effective; *X*-axis represents the willingness-to-pay for a QALY

**Fig. 3:**
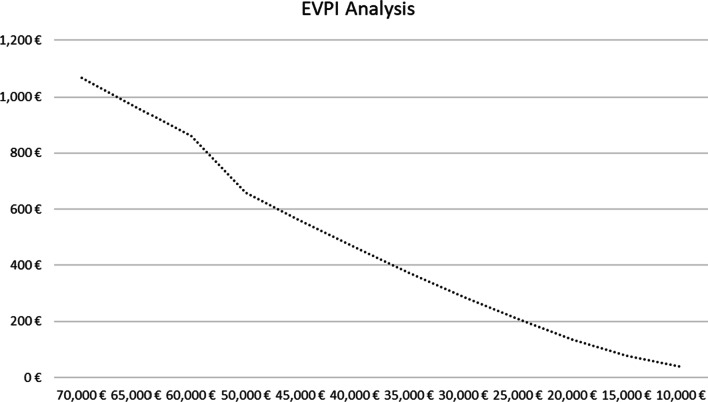
Analysis of expected value of perfect information. *Y*-axis represents the value of perfect information per patient; *X*-axis represents the willingness-to-pay for a QALY

It was assumed that the depicted ellipse followed the bivariate normal distribution, and its contour represented the 95% confidence intervals. Most bootstrap pairs fell into IV quadrant, in which PGx-guided treatment option is more effective and simultaneously less costly. Hence, there is a neutrality between the two alternatives in terms of QALYs as the 5000 dots were scattered almost evenly around the x- axis. In this aspect, since the concept of cost-effective represents a subjective assessment, a willingness-to-pay-threshold was determined to estimate the probability of acceptance or rejection of the PGx technology in the Spanish healthcare setting. Probabilistic results were illustrated using a cost-effectiveness acceptability curve (see Fig. 2) in which PGx-guided group (on the *y*-axis) may be cost-effective compared to control for a range (on the *x*-axis) of maximum monetary values that a decision-maker might be willing to pay per QALY. This explains that the acceptability curve is relatively independent of the value of the ceiling ratio and in favor of PGx technology. Indeed, the probability of PGx-guided treatment of being cost-effective increased significantly at a lower willingness-to-pay (WTP) threshold. Notably, at €50,000 per QALY, the probability of being cost-effectiveness was higher than 50%, at €30,000 was almost 62%, in case of WTP < €20,000, the probability overcome the 71%. EVPI analysis indicated that the cost of information for 50,000/QALY, 30,000/QALY and 20,000/QALY was determined at €659.4, €287.9 and €136.5, respectively (see Fig. 3).

## Discussion

Clopidogrel is the oldest and the most popular antiplatelet drug, used by millions of ACS patients every year. Besides its health benefits, it is demonstrated to be associated with increased risk of ADRs in a considerable number of patients. Apart from drug–drug interactions and the effect of concomitant medications, it was shown that individuals’ genotype affects clopidogrel metabolism leading to ADRs. *CYP2C19* genomic variants have been linked to variable response of CVD patients to clopidogrel, a fact that implies the necessity for more personalized treatment schemes following PGx testing.

In the present analysis, a cost-effectiveness analysis (CEA) of PGx-guided clopidogrel treatment in individuals suffering from different forms of ACS was conducted. This is one of the few studies that aims to compare the cost-effectiveness of PGx-guided treatment of this antiplatelet agent versus non-PGx-guided one in a cohort of Spanish patients with several comorbidities and without strict eligibility criteria. Our analysis concluded that PGx-guided treatment strategy cost two times less than conventional strategy and has a marginally higher effectiveness.

At first, it is noteworthy that this study is based on raw clinical and economic data derived from the PREPARE study which is the largest, multinational, controlled, cluster-randomized, crossover implementation study focusing exclusively in investigating the impact of preemptive PGx testing, a fact that differentiates it from most available studies in the literature that used simulated data [61–69]. This is very important since real-world evidence regarding each drug–gene pair is limited and RCT data are lacking population diversity and inclusion, a fact that raise concerns about data validity and health equality. PREPARE trial shares then a unique trial design that meets the scientific needs and can enhance the clinical significance of PGx.

As it was indicated, PGx-guided treatment represented a cost-saving option compared to a non-tailored one, sharing almost 50% less hospital admissions, less emergency visits and almost 13% less ADRs. All these clinical aspects imply an improvement in disease management that is also translated into costs’ reduction; PGx-guided treatment approach had 50% less total cost compared to the most conventional approach while most costs were related to hospitalization in both arms.

These important findings are congruent with the literature. Indeed, Fragoulakis and coworkers, demonstrated that in a cohort of Spanish patients undergoing percutaneous coronary intervention (PCI), PGx-guided treatment was dominant over standard of care with 0.9446 QALYs gained and €2971 cost compared to 0.9379 QALYs and €3205 at 1-year horizon [61]. Hospitalization was also the main type of costs and accounted for the majority of expenses for both arms [61]. Moreover, Claassens and coworkers in POPular Genetics concluded that a *CYP2C19* genotype-guided strategy was dominant over conventional therapy (prasugrel or ticagrelor) with 8.98 QALYs gained and €725 k cost savings in a simulated cohort of 1000 patients suffering from ACS [62]. In Dong and coworkers, it was shown that *CYP2C19* genotype-guided strategy was a cost-effective approach compared to a non-tailored one, on the grounds that it brought cost savings per patient (($4785/person vs. $5311/person) and a gain of 0.0027 QALYs [63]. The occurrence of ADRs was also reduced by 13% a fact that is in alliance with our findings and there was a significant decrease in medication costs by 20% [63]

Moreover, another study highlighted that in China, *CYP2C19* genotyping strategy to guide antiplatelet treatment was a cost-effective approach with an ICER of CNY 13,552.74 (US$1930.59) per QALY gained compared to standard of clinical care [64]. Probabilistic analysis demonstrated that in 95.7% of simulations, PGx-guided treatment was cost-effective in a WTP ranging from $0–$175,000 [64]. According to Reese and coworkers, in a simulated cohort of patients suffering from ACS, genotyping-driven group was the dominant treatment strategy owing to its higher clinical effectiveness and lower cost in comparison with universal prescription of clopidogrel to all patients, no matter their genetic makeup, while they focused on the number of adverse events prevented to express effectiveness [65].

In that study, calculated ICER was estimated at (ICER–$6,760, [95% CI –$6,720 to –$6,790]). In another study involving US patients conducted by Borse and coworkers, in which effectiveness was also measured in major cardiovascular events (MACE), it was indicated that PGx testing was cost-effective in 62% of the simulations when WTP threshold was set to US$ 50,000 while universal clopidogrel wasn’t [66]. Moreover, Limdi and coworkers in a simulated cohort demonstrated that PGx treatment was cost-effective ($42,365/QALY) and they pinpointed that it was more likely for PGx treatment to be cost-effective in different WTP thresholds in contrast to non-genotyping-driven strategies [67]. Moreover, in Singapore setting, Kim and coworkers concluded that genetic-driven treatment shared better QALYs and was less costly in the long-run, a conclusion that comes in accordance with Lala and coworkers [68, 69].

Following the results of a systematic review conducted by Verbelen and coworkers, in general most economic evaluations poses a positive attitude toward PGx-guided treatment [70]. More precisely, PGx-guided strategy was presented as dominant in 27% of published economic analysis while 30% of the studies concluded that PGx option is cost-effective [70]. Even if the Verbelen and coworkers study included publications of economic evaluations for all type of studies, it was highlighted that PGx-driven treatment was potentially a cost-effective option that could improve disease and drug management to great extent by diminishing healthcare expenditures [70].

In all above-mentioned studies [62–69], all data derived from literature or simulations and no raw data from clinical trials were used except of the Cai and coworkers study. In addition to it, both direct and induced costs were taken into consideration including medications and prescription costs while the analysis perspective was mainly those of payers (ie. sickness fund).

The superiority of PGx-guided treatment was also demonstrated in terms of less hospital admissions, less emergency visits and 50% reduction in ADRs occurrence and especially in those of high grade. All these features imply that PGx testing can offer a more optimal disease management and constitute a promising treatment strategy for ACS. These findings are in accordance with other studies. Reese and coworkers mentioned that tailoring one’s clopidogrel treatment following his/her genotyping results resulted in 450 less events [65]. In other words, one additional adverse event was prevented for every 23 individuals. This clinical endpoint can be translated in costs savings, less healthcare resources and less deaths. In a risk–benefit assessment, Guzauskas and coworkers came up with similar results as well [71].

They showed that PGx-guided treatment of ACS patients could reduce the incidence risk of suffering a health complication such as MI, stroke or death by 6.8% compared to universal clopidogrel use and decrease the risk of experiencing a MACE [66]. Given that clopidogrel is correlated with high incidence of MACE, the great difference between preemptive PGx testing and universal clopidogrel in terms of MACE incidence is very positive and illustrates the clinical significance of dosage adjustment, suggests that this approach could improve platelet inhibition and enhances the role of PGx implementation in drug and disease management.

Finally, according to the literature demographic factors such as age, BMI, and comorbidities such as obesity, diabetes and hypertension were proven to affect clopidogrel pharmacokinetics and drug metabolism. Indeed, Jiang and coworkers highlighted that there was scientific evidence regarding the association of age and prevalence of ADRs such as bleeding upon the use of clopidogrel, whereas it was shown that cardiac drugs belong to the top three classes of drugs that were responsible for even fatal ADR’s [8, 13]. Those factors may exert an impact on clinical effectiveness of clopidogrel, but it is shown to influence cost-effectiveness results. As reported by Nicolic and coworkers, age and gender had a slight affect in cost-effectiveness findings, but it wasn’t statistically significant while other parameters such as cardiac events could impact the analysis [47]. In this analysis, no demographic or individual characteristics had a significant influence in CEA. Only hypertension was indicated to affect ADR, a fact that comply with Nicolic and coworkers since hypertension was the only parameter with significant impact and not age or gender.

This study has a few limitations related to trial design. Not being a randomized controlled clinical trial in the strict sense, PREPARE comes with less strict inclusion/exclusion criteria and thus lower compliance. This fact led to low response rate and missing data, in terms of utility details. From the one hand, this fact seems to be an important drawback for study’s analysis, but from the other hand it is relatable to real-world data and represents a better cohort of patients that every clinician will meet during his/ her exercise.

## Conclusions

This CEA based on raw data from the PREPARE study in Spanish clinical sites suggests that preemptive genotyping before prescribing clopidogrel could add more value in the clinical practice and improve decision-making process for healthcare professionals. As genotyping was conducted as part of clinical care, unlike previous CEAs, our analysis was not limited by assumptions regarding the availability of genotype data in a timeline conducive for clinical care. Finally, PGx testing lowers the risk of ADRs occurrence and especially of life-threatening events and is possible to decrease the overall healthcare cost. Using raw clinical data that are closer to real-world situations gives an insight into the cohort of individuals eligible to receive clopidogrel treatment and the relevant costs. PGx will play an important role in CVD medicine by broaden the horizon for more efficient and cost-effective medications. Finally, all cost data (except of the genetic test price) derived from the official cost of Andalucía region representing sickness fund perspective, while a wider socioeconomic analysis could be the scope of the future research.

## Data Availability

The datasets used and/or analyzed during the current study are available from the corresponding author on reasonable request.

## Abbreviations

CVD: Cardiovascular disease
ACS: Acute coronary syndrome
MI: Myocardial infarction
PGx: Pharmacogenomics
ADR: Adverse drug reaction
eCRF: Electronic case report form
STEMI: ST elevation myocardial infarction
NSTEMI: Non-ST elevation myocardial infarction
DPWG: Dutch Pharmacogenomics Working Group
PM: Poor metabolizer
IM: Intermediate metabolizer
PREPARE: PREemptive Pharmacogenomic testing for preventing Adverse drug REactions [ADRs]
CCEIBA: Comité Coordinador de Ética de la Investigación Biomédica de Andalucía
QALY: Quality-adjusted life year
BMI: Body mass index
QOL: Quality of life
EVPI: Expected value of perfect information
GLM: Generalized linear model
WTP: Willingness-to-pay
MACE: Major cardiovascular event
SD: Standard deviation
UCI: Upper confidence interval
LCI: Lower confidence interval
CEA: Cost-effectiveness analysis
PCI: Percutaneous coronary intervention
